# Diagnostic challenges of masked arginine vasopressin deficiency in neurosarcoidosis complicated by sodium–glucose cotransporter 2 inhibitor use

**DOI:** 10.1210/jcemcr/luag318

**Published:** 2026-10-16

**Authors:** Ryohei Nonaka, Katsuhiro Tanaka, Akemi Tomimoto, Yukie Nagai, Takuya Nomura, Hirotaka Shibata

**Affiliations:** Department of Endocrinology and Metabolism, Oita Prefectural Hospital, Oita City, Oita 870-0855, Japan; Department of Endocrinology and Metabolism, Oita Prefectural Hospital, Oita City, Oita 870-0855, Japan; Department of Endocrinology and Metabolism, Oita Prefectural Hospital, Oita City, Oita 870-0855, Japan; Department of Endocrinology and Metabolism, Oita Prefectural Hospital, Oita City, Oita 870-0855, Japan; Department of Endocrinology and Metabolism, Oita Prefectural Hospital, Oita City, Oita 870-0855, Japan; Department of Endocrinology, Metabolism, Rheumatology and Nephrology, Faculty of Medicine, Oita University, Yufu City, Oita 879-5593, Japan

**Keywords:** neurosarcoidosis, hypercalcemia, hypopituitarism, arginine vasopressin deficiency, SGLT2 inhibitors

## Abstract

A wide variety of endocrine disorders caused by sarcoidosis have been reported to date. We report the case of an 82-year-old male patient who presented with hypercalcemia and anterior pituitary hormone deficiency at the time of his sarcoidosis diagnosis and was subsequently diagnosed with arginine vasopressin deficiency (AVP-D) 6 months after the initiation of glucocorticoid therapy. Magnetic resonance imaging revealed an enlarged pituitary gland, thickened pituitary stalk, and loss of the posterior pituitary bright spot, raising the suspicion of hypothalamic–pituitary sarcoidosis. Despite improvement in hypercalcemia, anterior pituitary function, and pituitary enlargement and stalk thickening with glucocorticoid therapy, subjective symptoms of dry mouth and polyuria gradually became apparent several months after the start of treatment. While the characteristic hypotonic polyuria of AVP-D was not prominent, we surmised that these typical clinical features were masked by adrenal insufficiency, chronic kidney disease (CKD), and concurrent use of a sodium–glucose cotransporter 2 (SGLT2) inhibitor for type 2 diabetes. This case highlights the fact that the typical presentation of AVP-D can be masked by concurrent SGLT2 inhibitor use and CKD, emphasizing careful assessment of water balance in such patients.

## Introduction

Sarcoidosis is a systemic granulomatous disease of unknown etiology that affects multiple organs. Neurosarcoidosis is observed in 5% to 15% of cases, and the diagnosis is often challenging due to the diversity of clinical symptoms and difficulty in histological diagnosis [1]. In particular, hypothalamic–pituitary (HP) sarcoidosis is an uncommon manifestation of neurosarcoidosis [2]. Symptoms of HP sarcoidosis include those of anterior pituitary insufficiency, arginine vasopressin deficiency (AVP-D), and hyperprolactinemia, whereas endocrine recovery after steroid therapy is uncommon [2]. This report describes a case of an elderly patient with suspected HP sarcoidosis who showed signs of improvement in anterior pituitary function after initiation of steroid therapy, but was subsequently diagnosed with AVP-D.

## Case presentation

An 82-year-old male patient receiving oral medication for type 2 diabetes and chronic kidney disease (CKD) visited his previous physician complaining of fatigue and loss of appetite for 2 weeks. Laboratory test results raised the suspicion of central hypothyroidism, leading to his referral to our hospital. His tongue was dry, indicating a tendency toward dehydration. Laboratory testing revealed low levels of thyroid-stimulating hormone (TSH) and free thyroxine (FT4). In addition, serum calcium levels were elevated (Table 1), leading to a diagnosis of hypercalcemia. The patient was admitted on the same day for further evaluation and treatment.

**Table 1:** Laboratory results on admission

Biochemistry	Value	Reference range
Sodium	132 mmol/L	138 to 145 mmol/L
Potassium	4.5 mmol/L	3.5 to 5.0 mmol/L
Chloride	99 mmol/L	101 to 108 mmol/L
Calcium	11.2 mg/dL (2.8 mmol/L)	8.5 to 10.2 mg/dL (2.12-2.54 mmol/L)
Inorganic phosphorus	4.1 mg/dL (1.3 mmol/L)	2.5 to 4.5 mg/dL (0.81-1.45 mmol/L)
Creatinine	1.88 mg/dL (166.2 μmol/L)	0.6 to 1.1 mg/dL (53-97 μmol/L)
eGFR	27.5 mL/min/1.73m^2^	≥60 mL/min/1.73m^2^
Albumin	3.2 g/dL (32 g/L)	4.0 to 5.2 g/dL (40-52 g/L)
Blood glucose	201 mg/dL (11.2 mmol/L)	70 to 99 mg/dL (3.9-5.5 mmol/L)
HbA1c	8.0% (64 mmol/mol)	4.9% to 6.0% (20-42 mmol/mol)
Plasma osmolality	286 mOsm/kg	275 to 290 mOsm/kg
Endocrinological data		
ACTH	13.0 pg/mL (2.86 pmol/L)	7.0 to 60.0 pg/mL (2.20-13.2 pmol/L)
Cortisol	6.94 μg/dL (191.5 nmol/L)	4.0 to 19.4 μg/dL (110-535 nmol/L)
TSH	0.172 μIU/mL (0.172 mIU/L)	0.5 to 5 μIU/mL (0.5-5 mIU/L)
FT3	2.33 pg/mL (3.58 pmol/L)	2.3 to 4.0 pg/mL (3.5-6.1 pmol/L)
FT4	0.83 ng/dL (10.7 pmol/L)	0.9 to 1.7 ng/dL (10-20 pmol/L)
PRL	52.9 ng/mL (1058 mIU/L)	4.29 to 13.69 ng/mL (100-600 mIU/L)
LH	<0.1 mIU/mL (<0.1 IU/L)	1.2 to 8.6 mIU/mL (1.2-8.6 IU/L)
FSH	1.58 mIU/mL (1.58 IU/L)	1.0 to 19.0 mIU/mL (1.0-19.0 IU/L)
Testosterone	<0.03 ng/mL (<0.1 nmol/L)	1.31 to 8.71 ng/mL (10-42 nmol/L)
GH	0.31 ng/mL (0.31 μg/L)	<2.47 ng/mL (<2.47 μg/L)
IGF-1	69 ng/mL (8.9 nmol/L)	41 to 163 ng/mL (5.3-21.2 nmol/L)
AVP	1.0 pg/mL (0.92 pmol/L)	<2.8 pg/mL (<2.6 pmol/L)
Calcium metabolism		
Intact PTH	4 pg/mL (0.42 pmol/L)	10 to 65 pg/mL (1.06-6.89 pmol/L)
PTHrP	<1.0 pmol/L	<1.1 pmol/L
1,25(OH)_2_ vitamin D_3_	47.4 pg/mL (115.7 pmol/L)	20 to 60 pg/mL (49.9-149.8 pmol/L)
Serology		
sIL-2R	1604 U/mL	120 to 500 U/mL
ACE	27.4 U/L	8.3 to 21.4 U/L
Urinalysis		
Specific gravity	1.014	1.002 to 1.03
Protein	(±)	
Glucose	(4+)	
Urine osmolality	415 mOsm/kg	50 to 1300 mOsm/kg

The reference range for IGF-1 is age-specific.

Abbreviations: ACE, angiotensin converting enzyme; ACTH, adrenocorticotropic hormone; AVP, arginine vasopressin; eGFR, estimated glomerular filtration rate; FSH, follicle-stimulating hormone; FT3, free triiodothyronine; FT4, free thyroxine; GH, growth hormone; HbA1c, glycohemoglobin; IGF-1, insulin-like growth factor 1; Intact PTH, intact parathyroid hormone; LH, luteinizing hormone; PRL, prolactin; PTHrP, parathyroid hormone–related protein; sIL-2R, soluble interleukin-2 receptor; TSH, thyroid-stimulating hormone.

## Diagnostic assessment

A computed tomography (CT) scan of the trunk revealed enlargement of the mediastinal and bilateral hilar lymph (BHL) nodes (Fig. 1A). Magnetic resonance imaging (MRI) revealed enlargement of the pituitary gland and pituitary stalk, as well as loss of the posterior pituitary bright spot (Fig. 1B). A mediastinal lymph node biopsy performed during bronchoscopy revealed a noncaseating epithelioid granulomatous pattern (Fig. 2), and the patient was therefore diagnosed with pulmonary sarcoidosis.

**Figure 1:**
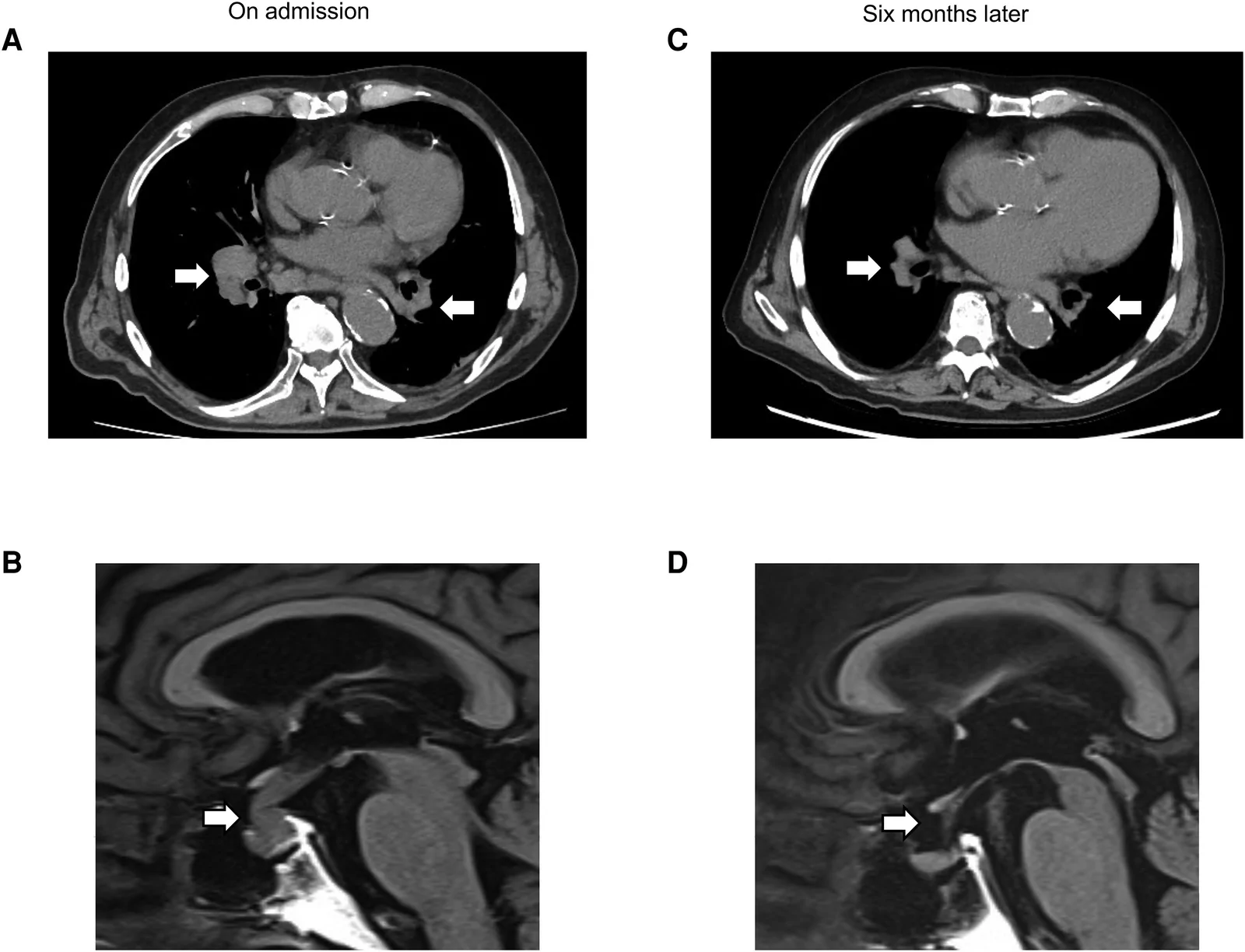
(A) Computed tomography (CT) scan of the trunk at admission showing bilateral hilar lymphadenopathy. (B) Sagittal T1-weighted magnetic resonance imaging (MRI) of the brain at admission revealed an enlarged pituitary gland, a thickened pituitary stalk, and loss of the posterior pituitary bright spot. (C) CT scan 6 months later showed improvement of the hilar lymphadenopathy. (D) MRI scan performed at approximately the same time demonstrated improvement of the pituitary enlargement and stalk thickening, but persistent loss of the posterior bright spot.

**Figure 2:**
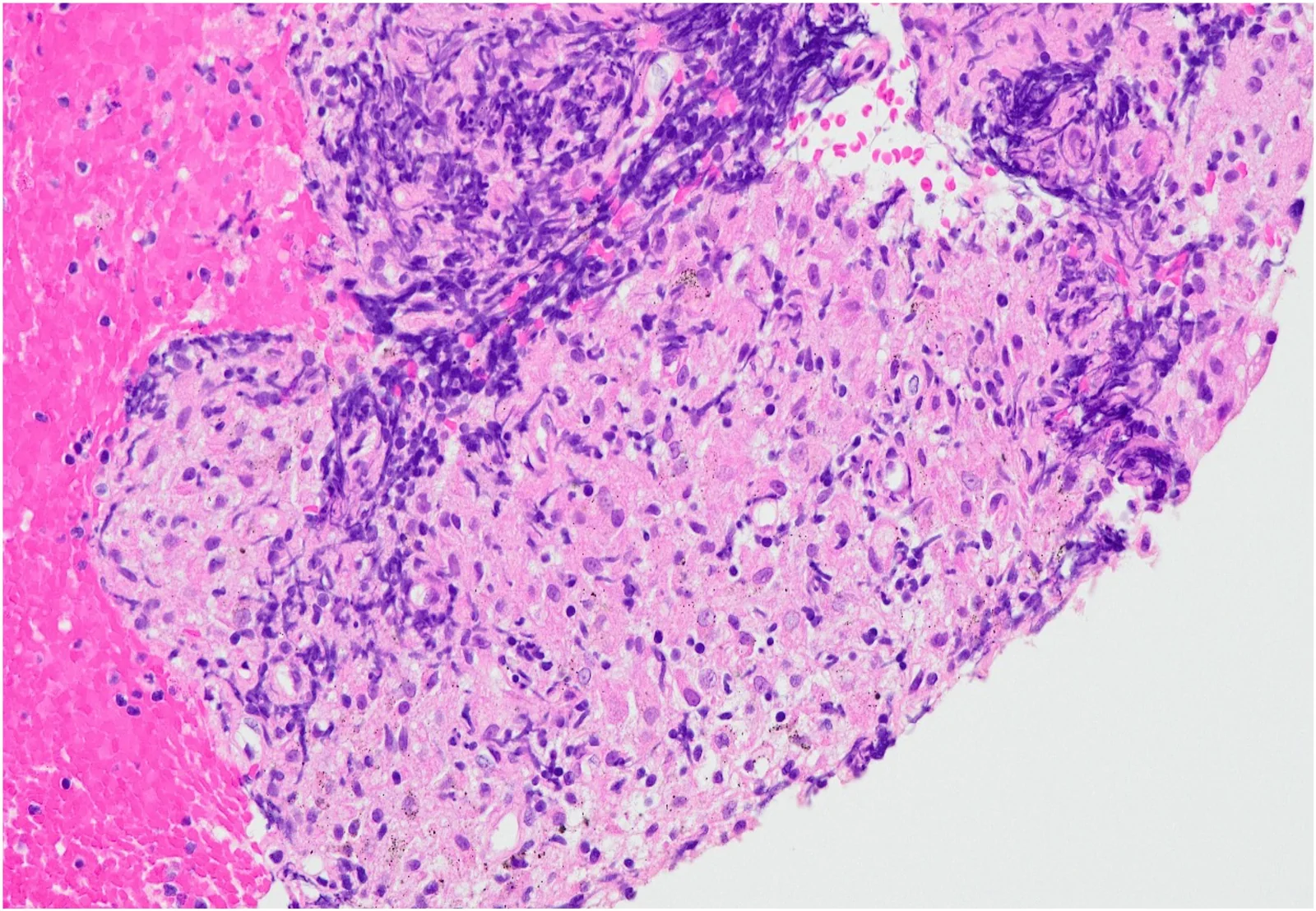
A lymph node biopsy specimen obtained via bronchoscopy showed noncaseating epithelioid granulomas (×200 magnification).

Endocrine function tests were performed because MRI findings raised the suspicion of HP sarcoidosis. Baseline pituitary hormone measurements revealed elevated prolactin (PRL), low luteinizing hormone (LH), and low TSH levels; there was no significant decrease in AVP levels. In the rapid adrenocorticotropic hormone (ACTH) stimulation test, the peak cortisol level was 16.6 μg/dL (International System of Units [SI]: 457.9 nmol/L) (reference range: ≥18 μg/dL [SI: ≥500 nmol/L]), indicating a subnormal response (Fig. 3A). In the corticotropin-releasing hormone (CRH) stimulation test, ACTH secretion was normal, but the cortisol response was poor (Fig. 3B). The responses of LH and TSH to gonadotropin-releasing hormone and thyrotropin-releasing hormone stimulation were reduced (Fig. 3C and 3D). Based on these results, the patient was diagnosed with secondary adrenal insufficiency, hypogonadism, and hypothyroidism.

**Figure 3:**
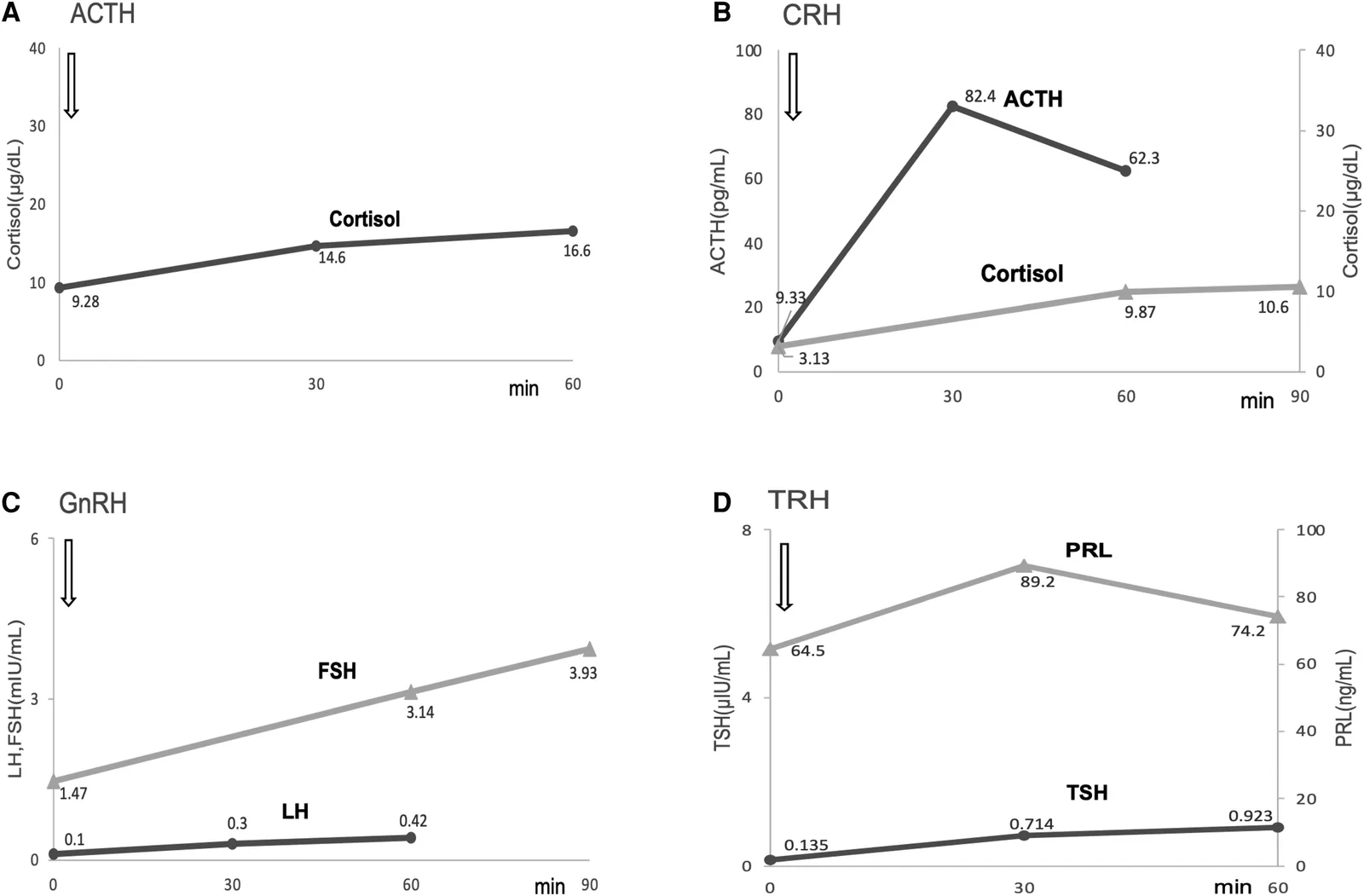
Endocrine function tests. (A) Decreased cortisol response to adrenocorticotropic hormone (ACTH) was observed. (B) The response of ACTH to corticotropin-releasing hormone (CRH) was preserved. (C) The secretion of luteinizing hormone (LH) was impaired, while follicle-stimulating hormone (FSH) secretion was preserved. (D) The secretion of thyroid-stimulating hormone (TSH) was impaired.

## Treatment

Figure 4 shows the patient's clinical course following hospitalization. Hypercalcemia was refractory to saline infusion and the calcitonin derivative elcatonin, but rapidly resolved after 35 mg/day of prednisolone therapy. The patient's fatigue and loss of appetite also improved. No symptoms suggestive of AVP-D, such as dry mouth, polydipsia, or polyuria, were observed before or after the initiation of glucocorticoid therapy. Next, 25 µg/day of levothyroxine was initiated for hypothyroidism. Since it was determined that the patient required rehabilitation, he was transferred to another hospital. Following discharge, the patient was followed up at the outpatient clinics of the departments of endocrinology and respiratory medicine.

**Figure 4:**
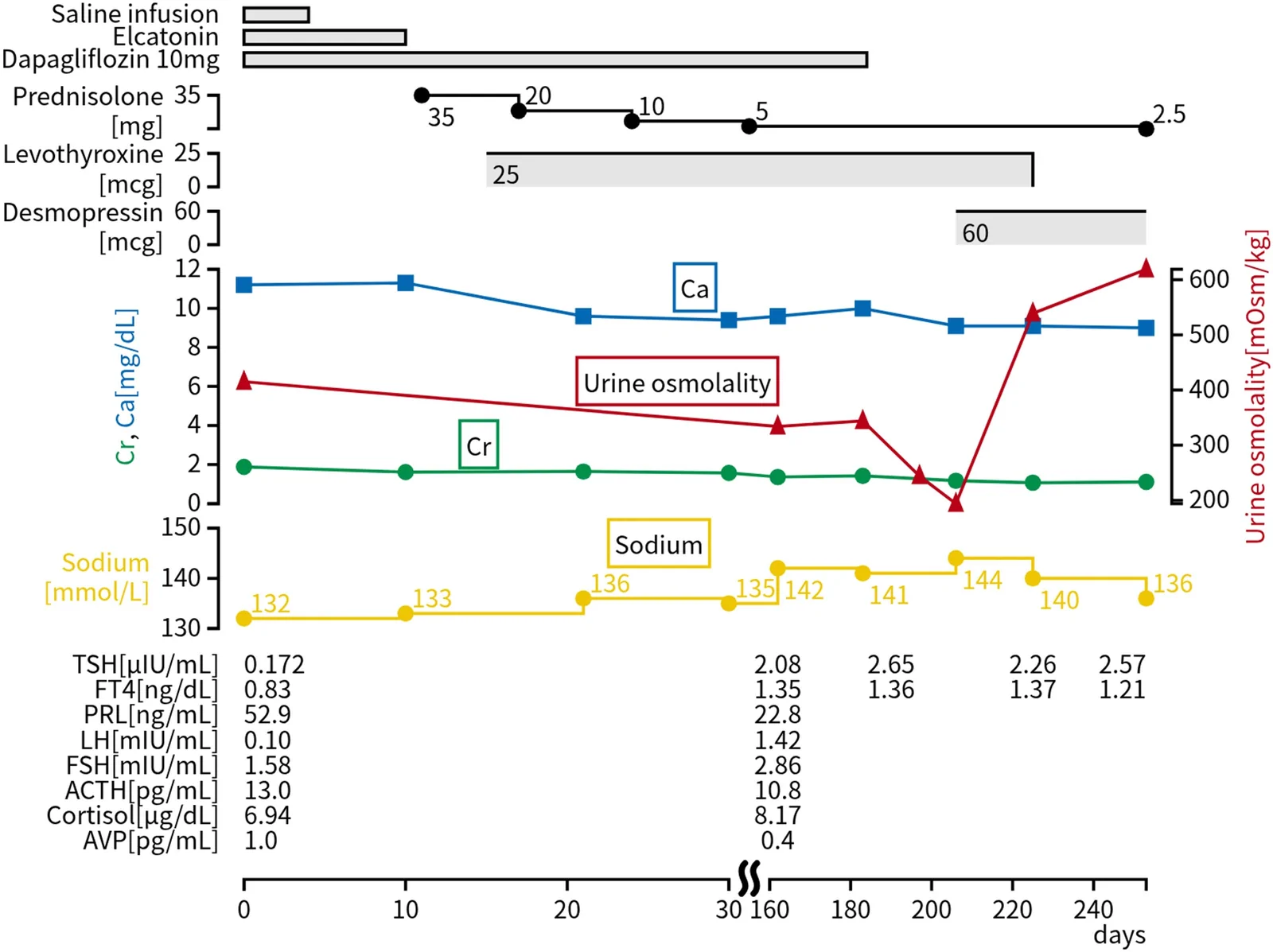
Clinical course. Changes in serum calcium, creatinine, urine osmolality, serum sodium, and hormone levels and medical treatments during hospitalization and outpatient follow-up.Abbreviations: Ca, calcium; Cr, creatinine; TSH, thyroid-stimulating hormone; FT4, free thyroxine; PRL, prolactin; LH, luteinizing hormone; FSH, follicle-stimulating hormone; ACTH, adrenocorticotropic hormone; AVP, arginine vasopressin.

## Outcome and follow-up

Subsequently, the prednisolone dosage was gradually tapered, with maintenance of serum calcium levels within the normal range. However, he began reporting symptoms of dry mouth and frequent urination several months later. A CT scan performed 6 months after the diagnosis of sarcoidosis showed improvement in the BHL (Fig. 1C). An MRI scan performed around the same time showed a decrease in the size of the pituitary gland and pituitary stalk, even though loss of the posterior pituitary bright spot persisted (Fig. 1D). Endocrinological findings showed a trend toward improvement in PRL, LH, and TSH levels (Fig. 4), although no significant changes were observed in ACTH and cortisol levels. At this time, although urinary osmolality was 334 mOsm/kg (reference range: 50-1300 mOsm/kg), showing no marked decrease, AVP levels were below the detection limit, raising the suspicion of AVP-D. The patient had been taking 10 mg/day of dapagliflozin for type 2 diabetes mellitus, but the drug was discontinued due to concerns regarding its effect on urine osmolality. Subsequently, urine osmolality decreased (244 mOsm/kg). At this time, his urine output ranged from 1400 to 2800 mL/day, and polyuria was not prominent. A blunted AVP response to a hypertonic (5%) saline load test was observed, and the desmopressin challenge test showed a decrease in urine volume and an increase in urine osmolality (Fig. 5). Based on these results, a diagnosis of AVP-D was made. The patient was started on oral desmopressin at 60 µg/day, and this treatment is still being continued as it has led to improvement in his symptoms of thirst and polyuria. Following initiation of desmopressin, serum sodium levels were closely monitored. No hyponatremia was observed during follow-up.

**Figure 5:**
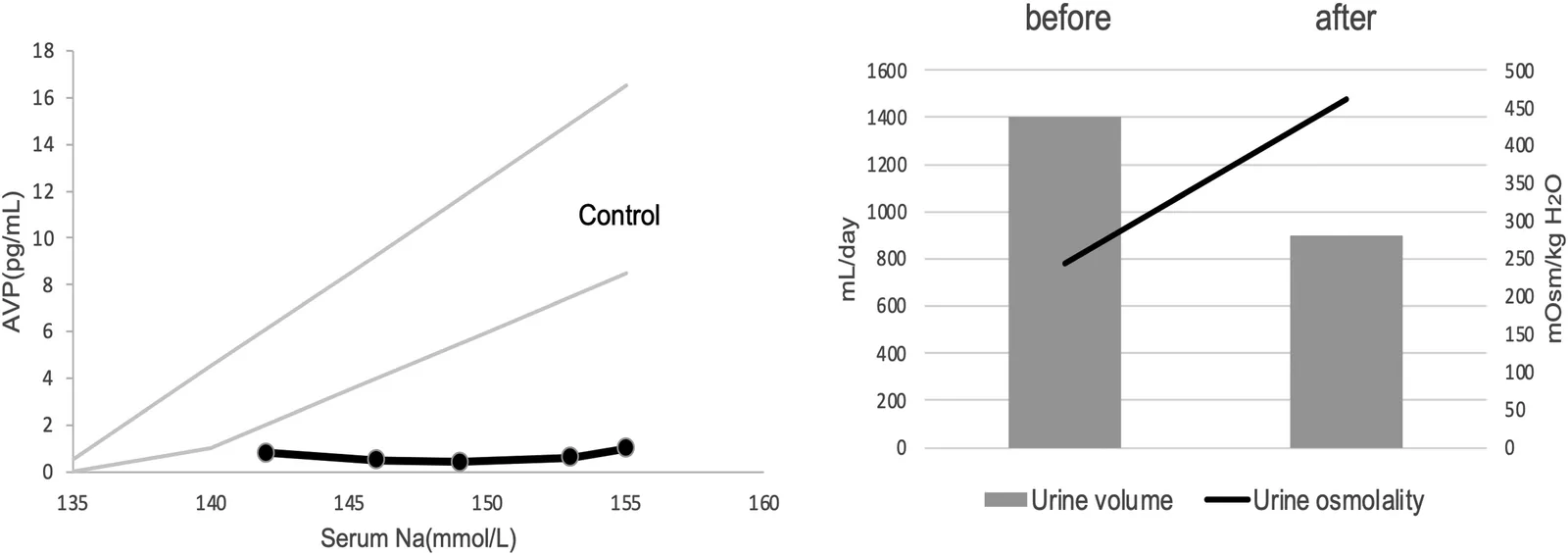
Hypertonic (5%) saline load test and desmopressin challenge test. The response of arginine vasopressin (AVP) to hypertonic saline infusion was reduced. Administration of AVP resulted in decreased urine volume and increased urine osmolality.

## Discussion

At the time of diagnosis of sarcoidosis in our case, hypopituitarism and hypercalcemia, considered to be attributable to the disease, were simultaneously observed. Hypopituitarism and hypercalcemia rarely occur simultaneously in sarcoidosis [3]. The patient's subjective symptoms, such as fatigue, were thought to be caused by both hypercalcemia and adrenal insufficiency. Endocrine disorders caused by neurosarcoidosis are triggered by dysfunction of the HP axis and are referred to as HP sarcoidosis [4]. The most common hormonal features are gonadotropin deficiency, followed by AVP-D, TSH deficiency, and hyperprolactinemia [5]. While imaging findings may improve with steroid therapy, endocrine dysfunction is often irreversible [5]. Based on the MRI findings and the presence of anterior pituitary insufficiency, HP sarcoidosis was considered highly likely. Although reports indicate that only 10% of cases of HP sarcoidosis show improvement in endocrine dysfunction despite treatment [6], in our case, baseline hormone levels of gonadotropins, TSH, and PRL showed a trend toward improvement, along with an improvement in imaging findings.

At the time of diagnosis of sarcoidosis, our patient did not report symptoms of dry mouth, polydipsia, or polyuria, and both serum and urine osmolality were normal. Therefore, the presence of AVP-D was initially ruled out. While there are previous case reports of masked AVP-D in which polydipsia and hypotonic polyuria became apparent immediately after initiating steroid therapy for sarcoidosis [7, 8], no such symptoms or findings were observed in our case even after treatment commencement. Although the patient reported subjective symptoms of dry mouth and polyuria 4 months later, a urine output exceeding 3 L/day, which would suggest AVP-D, was not observed. We hypothesized that the following 3 pathophysiological mechanisms were responsible for these observations. The first is adrenal insufficiency. Adrenal insufficiency impairs renal free water clearance and may also upregulate CRH secretion, which stimulates secretion of both AVP and ACTH [9]. Previous reports indicate that masked AVP-D due to adrenal insufficiency often develops within a few days of initiating glucocorticoid therapy [7, 8, 9]. Although our case did not develop polyuria immediately after glucocorticoid initiation, its partial masking by adrenal insufficiency at presentation cannot be excluded. The second is osmotic diuresis caused by sodium–glucose cotransporter 2 (SGLT2) inhibitors. Sodium–glucose cotransporter 2 inhibitors induce osmotic diuresis and may obscure hypotonic polyuria. This may mask a decrease in urine osmolality, potentially making the diagnosis of AVP-D difficult [8]. There have been reports of AVP-D cases in which hypotonic polyuria was not observed while the patient was receiving SGLT2 inhibitors, but became apparent after its discontinuation, leading to the diagnosis of AVP-D [10, 11]. Similarly, in our case, although urinary osmolality decreased after discontinuation of dapagliflozin (Fig. 4), no marked hypotonic polyuria was observed, as reported in previous studies. The third factor is a decrease in urinary dilution capacity due to CKD. Previous studies have shown that the ability to dilute urine becomes progressively impaired with advancing renal disease [12]. Chronic kidney disease reduces not only the ability to concentrate urine but also the ability to dilute it, due to factors such as disturbances in the interstitial hypertonicity of the renal medulla and impaired response of the principal cells of the concentrating nephron segments to vasopressin [12]. Furthermore, research has shown that minimum urine osmolarity increases while free water clearance decreases in CKD [13]. There have been reports of patients with end-stage renal failure developing polyuria immediately after receiving a kidney transplant, revealing the coexistence of AVP-D [14], and even in patients with moderate renal impairment, urine output may be reduced in the case of AVP-D. In our case, we hypothesized that the typical findings of AVP-D were masked by the combined effects of adrenal insufficiency, osmotic diuresis induced by the SGLT2 inhibitor, and reduced urine dilution capacity due to CKD. We believe that although SGLT2 inhibitors induce osmotic diuresis, the concurrent severe CKD and glucocorticoid deficiency suppressed the polyuric response. However, we cannot perform systematic daily urine-volume measurements due to the limitations of outpatient observation, and we cannot establish whether SGLT2 inhibitor and CKD directly contributed to urine volume in this case. Thus, the relative contributions of each factor to the absence of marked polyuria remain uncertain.

Most cases of AVP-D caused by HP sarcoidosis reported to date were irreversible [15]. Similarly, in cases of anterior pituitary insufficiency, functional recovery was often not achieved even when imaging findings improved, although cases with partial improvement in anterior pituitary function have also been reported [16]. This case was similar to ours in that glucocorticoid therapy improved anterior pituitary function along with the imaging findings, while AVP-D persisted. Since the severity and course of endocrine disorders caused by HP sarcoidosis vary widely, each individual case requires evaluation of hormonal secretion capacity, consideration of replacement therapy, and endocrinological follow-up. Furthermore, clinicians should recognize that the classical hypotonic polyuria of AVP-D can be masked in patients with underlying CKD or those taking SGLT2 inhibitors. When AVP-D is suspected in such clinical settings, careful assessment of water balance and a trial of discontinuing the SGLT2 inhibitor are warranted to establish an accurate diagnosis.

## Learning points

- Sarcoidosis presents with a wide range of symptoms and is closely associated with endocrine disorders, such as hypopituitarism and hypercalcemia.
- Hypopituitarism in HP sarcoidosis is often irreversible, but since functional improvement may occur, careful follow-up is essential.
- Multiple factors, such as glucocorticoid deficiency, advanced renal dysfunction, and SGLT2 inhibitor use, may contribute to the masked AVP-D.

## Contributors

All authors made individual contributions to authorship. R.N., K.T., A.T., Y.N., and T.N. were involved in the diagnosis and management of the patients. H.S. contributed to the analysis and interpretation of data and critical revisions of the manuscript for important intellectual content. All authors reviewed and approved the final draft.

## Data Availability

The original data generated and analyzed in this case report are included in this published article.
